# MRC2 Expression Correlates with TGFβ1 and Survival in Hepatocellular Carcinoma

**DOI:** 10.3390/ijms150915011

**Published:** 2014-08-26

**Authors:** Xiaohong Gai, Kangsheng Tu, Zhongtang Lu, Xin Zheng

**Affiliations:** Department of Hepatobiliary Surgery, the First Affiliated Hospital of Xi’an Jiaotong University, Xi’an, Shaanxi 710061, China; E-Mails: xhgai.xjtu@gmail.com (X.G.); tks0912@foxmail.com (K.T.); paulzheng@stu.xjtu.edu.cn (Z.L.)

**Keywords:** MRC2, hepatocellular carcinoma, TGFβ1, prognosis

## Abstract

MRC2 (Mannose Receptor C Type 2) is a constitutively recycling endocytic receptor belonging to the mannose receptor family, which has been found to be closely involved with cancer metastasis. This study attempted to determine MRC2 expression on hepatocellular carcinoma (HCC) and its significance on postsurgical prognosis of HCCs. The expression of both MRC2 and transforming growth factor (TGFβ1) was detected in tumor tissues and adjacent liver tissues from 96 HCCs by immunohistochemistry staining, and it was found that MRC2 expression in HCC tissues was significantly higher than in adjacent liver tissues. HCCs with higher MRC2 expression had worse prognosis after liver resection. Univariate analysis showed that advanced TNM staging of HCC, higher Edmonson-Steiner classification, intrahepatic metastases, portal vein invasion, higher MRC2 and higher TGFβ1 were the poor prognostic factors. Furthermore, multivariate analysis revealed that intrahepatic metastases, higher MRC2 and higher TGFβ1 were the independent prognostic factors. TGFβ1 treatment up-regulated MRC2 expression, cell migration and invasion of Huh7 cells notably. In addition, knockdown of MRC2 repressed the effect of TGFβ1 on cell migration and invasion. These data suggest that MRC2 overexpression predicts poor prognosis of HCCs after liver resection and MRC2 potentially contributed to TGFβ1-driven up-regulation of cell migration and invasion in HCC.

## 1. Introduction

Hepatocellular carcinoma (HCC) is one of the highly prevalent malignant diseases and is the third leading cause of cancer related death all through the world [1]. Incidences of the disease continue to increase year after year [2]. Because there are no typical clinical manifestations and high metastasis capacity at the early stage, most HCC patients cannot receive curative surgical treatments including radical liver resection and liver transplantation. Additionally, due to its high recurrence and metastasis characteristics, the prognosis of HCC after hepatic resections including curative one and palliative one is dismal. Therefore, there is an urgent need to figure out the underlying molecular mechanism of high metastasis capacity of HCC and explore the novel markers predicting HCC metastasis efficaciously in clinic.

Cancer metastasis is a kind of tissue remodeling process in which normal tissue in the local organ and or distant organs is invaded and eventually completely replaced by cancer tissues [3]. The deposition and degradation of the extracellular matrix are the critical features of cancer metastasis. Among them, there are multiple functions of extracellular matrix degradation on tumor progression including: (1) providing tumor mass more space to spread; (2) liberating more latent growth factors from extracellular matrix and activating oncogenic cell signalings in neighboring tumor cells; (3) facilitating the formation of neovasculature within tumor mass; (4) abolishing the restrictions of tumor cell proliferation from extracellular matrix [4]. Thus, it is crucial to demonstrate the mechanism of extracellular matrix degradation.

Transforming growth factor (TGFβ1) is a pleiotropic cytokine which contributes to wound healing, angiogenesis, fibrosis and cancer [5]. It was first identified in mammary epithelial development by Daniel and his colleagues in 1987 [6]. A growing body of evidences showed that TGFβ1 functions as either a tumor suppressor or a tumor promoter [7,8,9,10,11,12] via both SMAD (drosophila mothers against decapentaplegic protein)-dependent and SMAD-independent cascades. In fact, the multifunctional activities of TGFβ1 depends on the stage of carcinogenesis. In normal epithelia, it acts as tumor repressors through both inhibiting cell proliferation and inducing apoptosis, whereas accelerating progression of established cancers via activating various oncogenic cell signals and inducing EMT directly [11,13,14]. Generally, the tumor promoter function of TGFβ1 in the context of cancer progression results from two actions including losing its inhibitory effect on carcinogenesis and awaking its oncogenic function. Therefore, it is important to explore the key factors which play an important roles in the conversion of TGFβ1 function from a tumor suppressor to a tumor promoter.

Mannose Receptor C Type 2 (MRC2), also known as uPARAP/Endo180, is a constitutively recycling endocytic receptor belonging to the mannose receptor family [15]. Through the endocytic function, MRC2 is found to bind and internalize both intact and degraded collagens and in turn take part in the turnover of collagens in both cytomembrane and extracellular matrix [16]. Recently, there have been accumulating reports showing that MRC2 is aberrantly up-regulated in a variety of cancers and closely involved in cancer metastasis including breast cancer [17,18], prostate cancer [19], head stroma, and neck cancer [20]. However, the expression of MRC2 and its role in HCC remain unclear. In this study, we attempted to address the following questions: (1) Is MRC2 expression up-regulated aberrantly in HCC tissues compared to adjacent liver tissues? (2) What is the relationship between MRC2 expression in HCC tissues and HCC prognosis after liver resection? (3) What is the correlation between TGFβ1 and MRC2? (4) Is MRC2 attributed to the oncogenic effect of TGFβ1 in HCC?

## 2. Results and Discussion

### 2.1. Mannose Receptor C Type 2 (MRC2) Was Over-Expressed in Hepatocellular Carcinoma (HCC) Tissues and Associated with Poor Prognosis after Liver Resection

**Figure 1:**
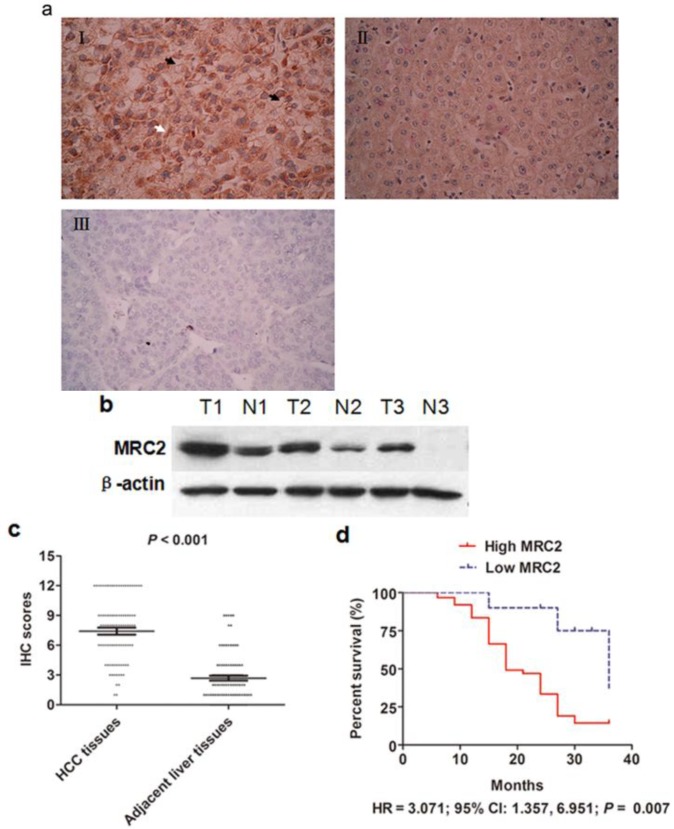
(**a**) (**I**) The representative immunohistochemistry (IHC) staining of MRC2 in HCC tissues. MRC2 protein located in both cell membrane (black arrow) and cytoplasm (white arrow); (**II**) The representative IHC staining of MRC2 in adjacent liver tissues; (**III**) The negative staining of MRC2 in HCC tissues; (**b**) The western immunoblotting results of MRC2 in 3 pairs of representative HCC tissues (T) and adjacent liver tissues (N); (**c**) The IHC scores of MRC2 in HCC tissues were significantly higher than one in adjacent liver tissues (*p* < 0.001); and (**d**) The comparison of Kaplan–Meier survival curves showed that patients with higher MRC2 expression in HCC tissues had worse prognosis after liver resection (HR = 3.071; 95% CI: 1.357, 6.951; *p* = 0.007).

To observe MRC2 expression in HCC, immunohistochemistry (IHC) staining assays were carried out and it was found that MRC2 protein located in both cell membrane and cytoplasm, as shown in Figure 1a. By analyzing the IHC score using the Mann–Whitney U test, we found that MRC2 expression in HCC tissues was significantly higher than one in adjacent liver tissues (*p* < 0.001; Figure 1c). We also examined MRC2 expression by western immunoblotting in 3 pairs of representative HCC tissues and adjacent liver tissues. As shown in Figure 1b, there was significantly more MRC2 expression in HCC tissues than in adjacent liver tissues. The relationship between MRC2 and the clinicopathological parameters of the 96 HCCs was statistically assessed and the results were listed in Table 1. The expression of MRC2 was significantly associated with intrahepatic metastases (*r* = 0.485, *p* = 0.005) and portal vein invasion (*r* = 0.214, *p* = 0.002). However, there was no significant correlation between MRC2 expression in HCC tissues and gender (*r* = −0.16, *p* = 0.062), age (*r* = 0.344, *p* = 0.121), HBV infection (*r* = 0.312, *p* = 0.075), liver cirrhosis (*r* = 0.12, *p* = 0.063), serumα-fetoprotein (AFP) level (*r* = 0.322, *p* = 0.130), tumor size (*r* = 0.271, *p* = 0.091), Edmonson–Steiner classification (*r* = 0.332, *p* = 0.130) and vasculature invasion (*r* = 0.070, *p* = 0.071).

**Table 1:** Correlation between the Clinicopathological Parameters and Mannose Receptor C Type 2 (MRC2) Expression in 96 HCCs.

Clinicopathological Parameters		No. of Higher MRC2 Expression in HCC Tissues	*r*-Value	*p*-Value
Gender	Female (*n* = 39)	28	−0.16	0.062
	Male (*n* = 57)	34		
Age	>50 (*n* = 49)	43	0.344	0.121
	<50 (*n* = 47)	19		
HBV Infection	Yes (*n* = 76)	59	0.312	0.075
	No (*n* = 20)	3		
Liver Cirrhosis	Yes (*n* = 89)	61	0.12	0.063
	No (*n* = 7)	1		
High Serum AFP Level	>400 ng/mL	38	0.322	0.130
	<400 ng/mL	24		
Tumor Size	>5 cm	40	0.271	0.091
	<5 cm	22		
Intrahepatic Metastases	Yes (*n* = 18)	16	0.485	0.005
	No (*n* = 78)	46		
Portal Vein Invasion	Yes (*n* = 9)	9	0.214	0.002
	No (*n* = 87)	53		
Portal Vein Invasion	Yes (*n* = 9)	9	0.214	0.002
	No (*n* = 87)	53		
Edmonson–Steiner Classification	I and II (*n* = 34)	24	0.332	0.130
	III and IV (*n* = 62)	38		
Advanced TNM Stage	I and II (*n* = 52)	26	0.151	0.032
	III and IV (*n* = 44)	36		
Vasculature Invasion *	Yes (*n* = 12)	7	0.070	0.071
	No (*n* = 84)	55		

* Vasculature invasion means microscopic vein invasion; AFP: serumα-fetoprotein; TNM: TNM Classification for Hepatocellular Carcinoma.

We obtained the follow-up information from 72 of 96 HCC cases (75%). The median time of follow-up was 18 months. We compared the Kaplan-Meier survival curves between the high MRC2 group, in which HCC tissues had more MRC2 expression than adjacent liver tissues, and in the low MRC2 group and found that the high MRC2 group had clearly worse prognosis after liver resection than the low MRC2 group (HR = 3.071; 95% CI: 1.357, 6.951; *p* = 0.007; Figure 1d), which pointed out that MRC2 over-expression in HCC tissues was an important factor associated with poor outcome after liver resection. After univariate analysis, it was found that advanced TNM staging, higher Edmonson–Steiner classification, intrahepatic metastases, portal vein invasion, higher MRC2 and higher TGFβ1 were the poor prognostic factors. Furthermore, multivariate analysis revealed that intrahepatic metastases, higher MRC2 and higher TGFβ1 were the independent prognostic factors for HCC patients after liver resection (as shown in Table 2).

**Table 2:** Cox proportional-hazard regression analysis of the relationship between clinicopathologic parameters and overall survival rate of HCC Patients after liver resection.

Clinicopathologic Parameters	Unvariate Analysis		Multivariate Analysis	
	RR (95% CI)	*p*-Value	RR (95% CI)	*p*-Value
Intrahepatic Metastases	3.120 (2.132–6.324)	0.001	2.975 (1.587–4.525)	0.003
Higher MRC2 Expression in HCC Tissue	1.856 (1.323–2.241)	0.012	2.035 (1.785–2.995)	0.015
Higher TGFβ1 Expression in HCC Tissue	1.697 (1.022–1.975)	0.030	1.882 (1.211–2.214)	0.022

### 2.2. Transforming Growth Factor (TGFβ1) Was Up-Regulated in HCC Tissues and Associated with MRC2 Positively

**Figure 2:**
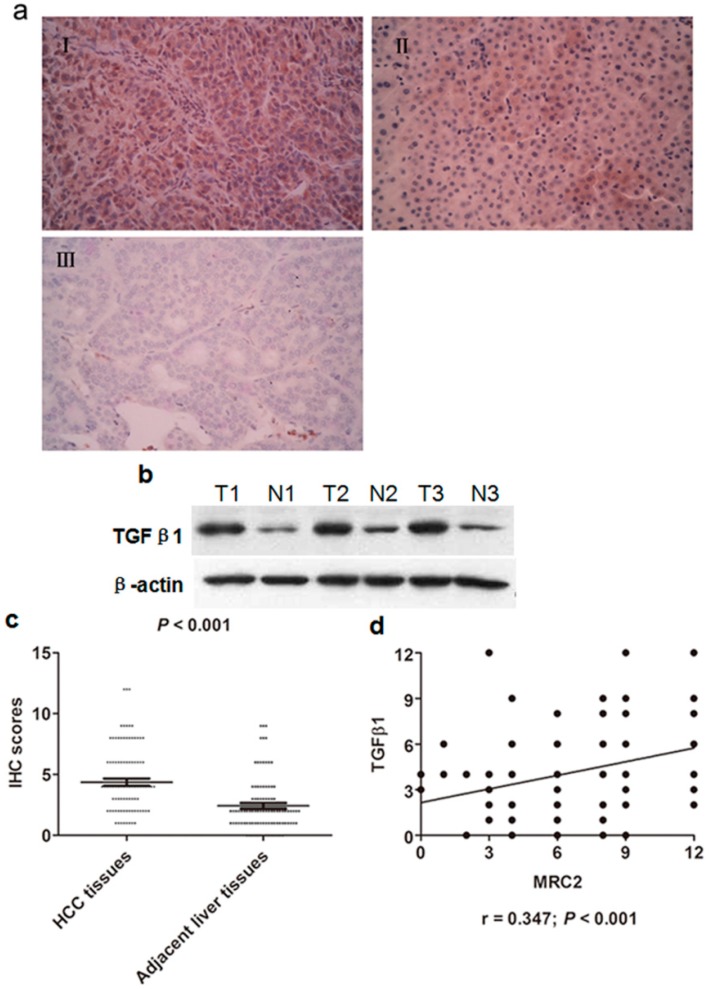
(**a**) (**I**) The representative IHC staining of Transforming growth factor (TGFβ1) in HCC tissues; (**II**) The representative IHC staining of TGFβ1 in adjacent liver tissues; (**III**) The negative staining of TGFβ1 in HCC tissues; (**b**) The western immunoblotting results of TGFβ1 in 3 pairs of representative HCC tissues (T) and adjacent liver tissues (N); (**c**) The IHC scores of TGFβ1 in HCC tissues were significantly higher than one in adjacent liver tissues (*p* < 0.001); (**d**) The Spearmen rank test showed that there was positive correlation between TGFβ1 and MRC2 expression in HCC tissues (*r* = 0.347; *p* < 0.001).

To determine the relationship between TGFβ1 and MRC2 in HCC tissues, we also observed TGFβ1 expression in both HCC tissues and adjacent liver tissues by IHC staining. As shown in Figure 2a, the majority of positive TGFβ1 staining cells showed diffused cytoplasmic staining. After analyzing the IHC scores by the Mann–Whitney test, we found that the expression of TGFβ1 in HCC tissues was apparently more than one in adjacent liver tissues (*p* < 0.001; Figure 2c). Additionally, expression of TGF β1 in the representative HCC tissues was found apparently more than that in the matching adjacent liver tissues (shown in Figure 2b). The Spearmen rank test also verified that there was a significantly positive correlation between TGFβ1 and MRC2 expression in HCC tissues (*r* = 0.347, *p* < 0.001; Figure 2d), which initially indicated that TGFβ1 could be involved in aberrant over-expression of MRC2 in HCC.

### 2.3. TGFβ1 Treatment Up-Regulated MRC2 Expression and Enhanced Cell Mobility and Invasion of HCC Cells

**Figure 3:**
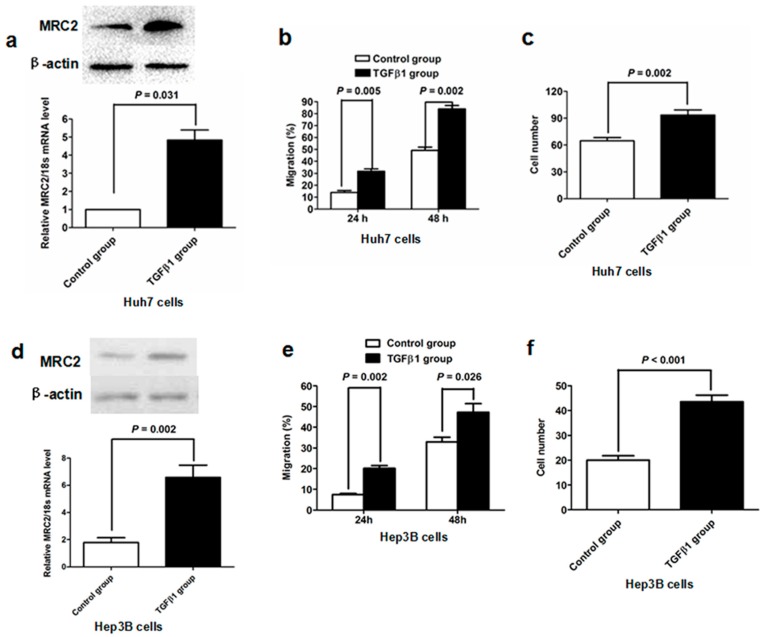
(**a**) As assessed by both western immunoblotting and qRT-PCR assays, TGFβ1 treatment leaded to up-regulation of MRC2 in Huh7 cells significantly; (**b**) TGFβ1 treatment promoted migration of Huh7 cells apparently; (**c**) Invasion capacity of Huh7 cells was significantly enhanced by TGFβ1 treatment (*p* = 0.002); (**d**) Both qRT-PCR and western immunoblotting revealed that TGFβ1 treatment resulted in a significant increase of MRC2 expression in Hep3B cells; (**e**) The migration ability of Hep3B cells was increased by TGFβ1 treatment obviously; (**f**) Millicell invasion chamber assay showed that TGFβ1 treatment up-regulated invasion ability of Hep3B cells clearly.

To examine further whether TGFβ1 take part in aberrant MRC2 over-expression in HCC, we treated Huh7 cells with recombinant human TGFβ1 protein *in vitro*. It was found that MRC2 expression of Huh7 cells was increased at the level of both mRNA and protein 48 h after TGFβ1 treatment at the concentration of 10 ng/mL (Figure 3a). The scratch wound healing migration assay showed that the migration rate of Huh7 cells stimulated by TGFβ1 (TGFβ1 group) was obviously higher than that of control Huh7 cells (Control group) at 24 and 48 h after scratching (*p* = 0.005 and *p* = 0.002, respectively; Figure 3b). In the invasion assay, there were 94 ± 6 Huh7 cells per field crossing the Matrigel gel and chamber filter in TGFβ1 group, whereas 65 ± 4 Huh7 cells per field in Control group (*p* = 0.002; Figure 3c). To further verify the regulatory function of TGFβ1 on MRC2 expression in HCC cells, we treated Hep3B cells with TGF β1. Similarly, MRC2 expression was found to be increased apparently by both qRT-PCR and western immunoblotting, as shown in Figure 3d. The migration and invasion capacities of Hep3B cells were also promoted by TGFβ1 treatment (Figure 3e,f). These data strongly supported that TGFβ1 promoted tumor cell mobility and invasion and meanwhile induced up-regulation of MRC2 in HCC cells.

### 2.4. Knockdown of MRC2 Abolished the Effect of TGFβ1 on Cell Mobility and Invasion

**Figure 4:**
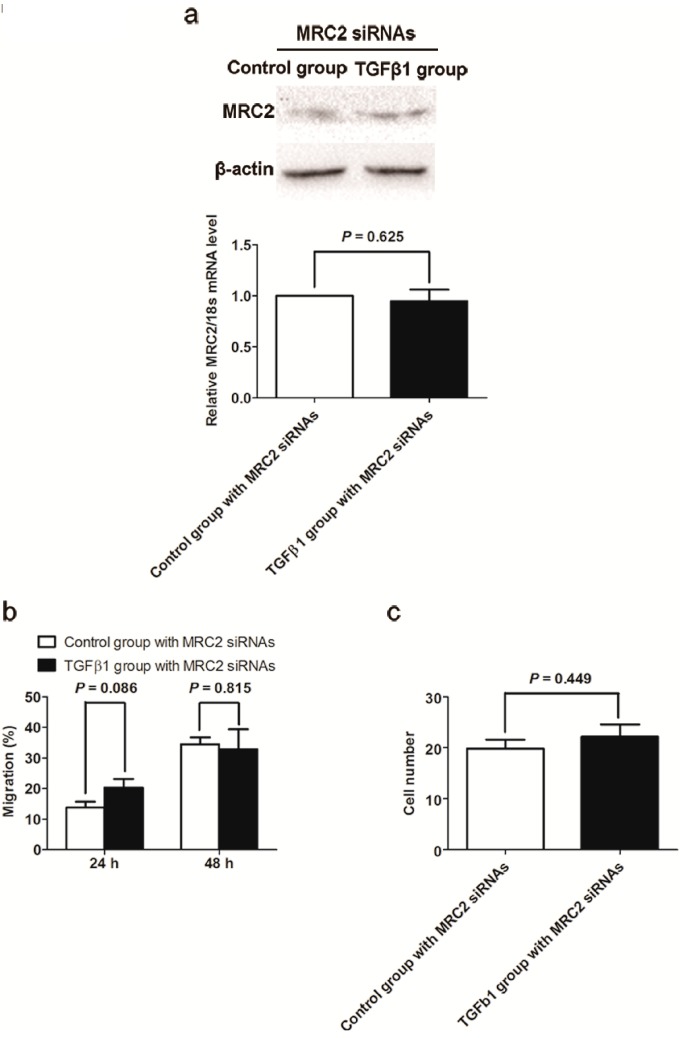
(**a**) As assessed by both western immunoblotting and qRT-PCR assays, TGFβ1 treatment leaded to up-regulation of MRC2 in Huh7 cells significantly; (**b**) TGFβ1 treatment promoted migration of Huh7 cells apparently; and (**c**) Invasion capacity of Huh7 cells was significantly enhanced by TGFβ1 treatment (*p* = 0.001).

To investigate whether MRC2 plays an important role in the oncogenic function of TGFβ1, we transfected MRC2 siRNA into Huh7 cells one day before TGFβ1 treatment in order to repress MRC2 up-regulation induced by TGFβ1. The qRT-PCR and western immunoblotting assays both confirmed that there was not a significant increase in MRC2 expression of Huh7 cells after TGFβ1 treatment (Figure 4A). As shown in Figure 4B, after suppression of MRC2 up-regulation in Huh7 cells, there was no significant difference on cell migration rate between TGFβ1 group and Control group found at 24 and 48 h after scratching (*p* = 0.086 and *p* = 0.815, respectively). Consistently, the number of cell crossing both the Matrigel gel and chamber filter in TGFβ1 group was similar with that in Control group, as assessed by the invasion assay (*p* = 0.449; Figure 4C). Hence, it was demonstrated by these data that TGFβ1 couldn’t promote mobility and invasion ability of Huh7 cells without MRC2.

### 2.5. TGFβ1/Smad3 Pathway Regulated MRC2 Expression in HCC Directly

**Figure 5:**
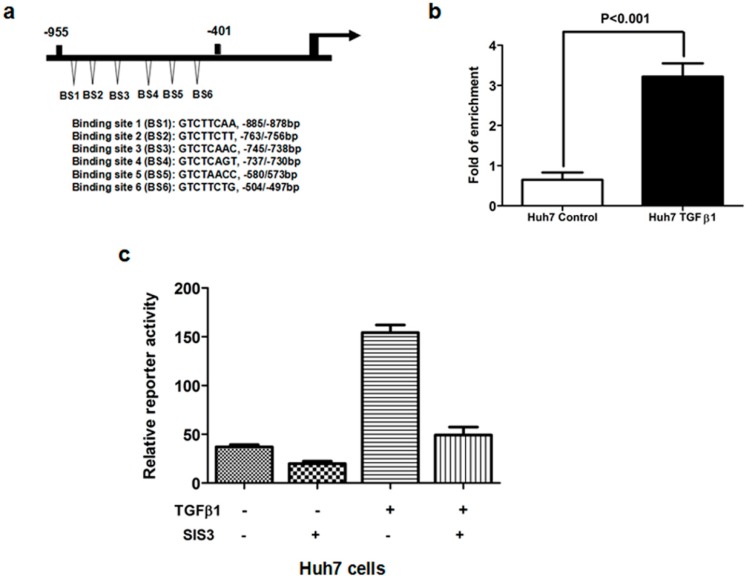
(**a**) Schematic diagram of the potential Smad binding sites in the promoter of MRC2 which shows there are six neighboring potential Smad binding sites in the −955/−401 bp MRC2 promoter fragment; (**b**) With the help of ChIP assay, we obtained the DNA fragments bound with Smad protein in the nucleus of Huh7 cells. As assessed by PCR assay, TGFβ1 treatment increased significantly the occupancy of Smad protein in the MRC2 promoter; (**c**) The luciferase reporter assay showed that TGFβ1 treatment leaded to more MRC2 luciferase activity in Huh7 cells, which was abolished by the Smad3 inhibitor SIS3. These data strongly demonstrated that Smad3 was involved closely in the regulatory effect of TGFβ1 on MRC2 expression.

To reveal the role of TGFβ1 on the transcriptional regulation of MRC2 in HCC, we identified several potential Smad binding sites in the gene promoter of MRC2 using MatInspector professional version 7.2 (shown in Figure 5a). The chromatin immunoprecipitation (ChIP) assay was carried out to test the direct interaction between Smad and the MRC2 promoter in Huh7 cells treated with TGFβ1 and confirmed that Smad protein was bound with the promoter of *MRC2* gene in the nucleus of HCC cells, as shown in Figure 5b. Hence, the −955/−401 bp MRC2 promoter fragment was initially identified to contain the Smad binding sites and the −955/−1 bp fragment reconstituted into the luciferase reporter vector, pGL3-basic. To address whether Smad3 contributes to the regulatory effect of TGFβ1 on MRC2 expression, we treated Huh7 cells with SIS3, a kind of Smad3 inhibitor. As shown in Figure 5c, SIS3 treatment resulted in an apparent decrease in MRC2 promoter activity in the presence of TGFβ1, as assessed by luciferase report assay. Thus, these data strongly supported that TGFβ1/Smad3 signaling up-regulated the expression of MRC2 in HCC cells via mediating positively its transcription directly.

## 3. Experimental Section

### 3.1. Patients and Specimens

There were a total of 96 HCC patients recruited in this investigation which underwent liver resection in our hospital from 2009 to 2010. HCC tissues and adjacent liver tissues (>2 cm distance to the resection margin) were obtained and kept in paraformaldehyde immediately. The diagnosis of all patients was confirmed by pathologists. Not all patients did received chemotherapy or embolization therapy before surgery. Demographic, clinical and histopathological information were collected from the medical records and shown in Table 1. The follow-up was carried out by our group to analyze postsurgical over-all survival of HCCs. Written informed consent was obtained from all patients recruited in this study. The ethics committee of the First Affiliated Hospital of Xi’an Jiaotong University approved all protocols according to the Helsinki Declaration of 2013 (No.20080301, 6 May 2008).

### 3.2. Immunohistochemistry Staining

Paraffin-embedded tissue sections were subjected to immunohistochemical analysis as described previously [21]. The primary rabbit anti-MRC2 antibody (Catalog No.: ab70132) and primary rabbit anti-TGFβ1 antibody (Catalog No.: ab92486) were both obtained from Abcam (Cambridge, MA, USA). The slides were dewaxed and dehydrated. After rehydration, endogenous peroxidase activity was blocked for 30 min using a methanol solution containing 0.3% hydrogen peroxide. After antigen retrieval in citrate buffer, we blocked all slides at 4 °C overnight with 2% BSA. The slides were incubated respectively with the primary antibodies targeting MRC2 and TGFβ1 overnight at 4 °C. Then the slides were washing with PBS three times and incubated with the biotinylated secondary antibody which was purchased from Zhongshan Goldenbridge Biotechnology Limited Company (Beijing, China) according to the manufacturer’s instruction. We stained the slides by the avidin–biotin–peroxidase complex (SABC) method. The slides were visualized with diaminobenzidine and counterstained with hematoxylin. Finally, tissues were dehydrated in alcohol and xylene.

**Figure 6:**
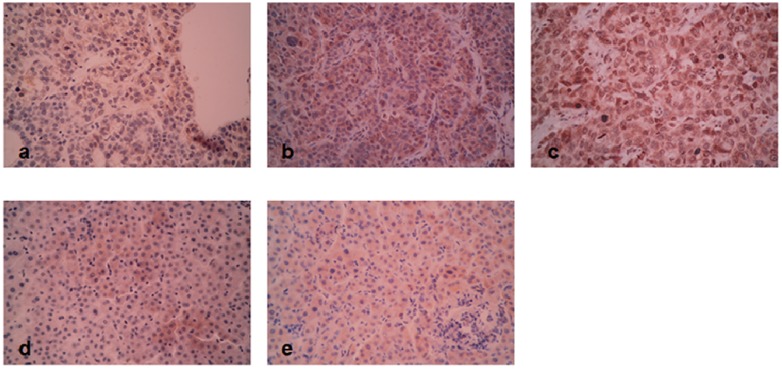
(**a**) The representative MRC2 staining of HCC tissue with 1 score; (**b**) the representative MRC2 staining of HCC tissue with 6 score; (**c**) the representative MRC2 staining of HCC tissue with 12 score; (**d**) the representative MRC2 staining of adjacent liver tissue with 1 score; (**e**) the representative MRC2 staining of adjacent liver tissue with 6 score.

Two experienced pathologists observed all slides independently to assess the Edmonson classification clinical TNM grading, maximum tumor diameter and the results of IHC assay. The results of IHC staining were evaluated by the staining intensity and the percentage of specifically positive staining tumor cells. Staining intensity was classified into four grades: 0, none; 1, weak; 2, moderate; 3, strong. The percentage of specifically positive staining tumor cells was given with the following grades: 0 (<5%), 1 (6%–25%), 2 (26%–50%), 3 (51%–75%), and 4 (>75%). The IHC staining score was expressed by multiplying the staining intensity and the percentage of specifically positive staining tumor cells. The IHC score of >1 was considered as positive staining. Each slide was observed for 10 independent high magnification field (×400) to get the mean staining score. The representative figures of each score were shown in Figure 6.

### 3.3. Cell Culture and TGFβ1 Treatment

Huh7 cell line was a kind gift from Kefeng Dou (Department of Hepatobiliary Surgery, Xijing Hospital, Fourth Military Medical University, Xi’an, China) and cultured with DMEM medium with 10% FBS at 37 °C in a humidified atmosphere of 95% air and 5% CO_2_. For TGFβ1 treatment, Huh7 cells were cultured with 1% FBS for 24 h and stimulated with 10 ng/mL of TGFβ1 for 48 h.

### 3.4. RNAi Transfections

We purchased the siRNAs against MRC2 (Catalog No.: sc-62276) and the scrambled siRNAs (Catalog No.: sc-37007) from Santa Cruz Biotechnology (Santa Cruz, CA, USA). Huh7 cells were plated in six-well plates at the concentration of 0.2 × 106 per well and cultured overnight. Then Huh7 cells in each well were transfected with 100 nM siRNAs using Lipofectamine RNAi MAX Reagent (Invitrogen, CA, USA) according to the manufacture’s recommendations. The cells were used for further experiments at 48 h after transfection.

### 3.5. Western Immunoblotting

Western immunoblotting assay was carried out as described previously [22]. Briefly speaking, 30 mg denatured protein samples were separated by polyacrylamide gel electrophoresis (PAGE) and then transferred into PVDF membrane. After blocked with 10% BSA in tris-buffered saline buffer (TBS) for 4 h at room temperature, the blots in PVDF membrane were incubated overnight with the primary antibodies respectively at 4 °C. After washed 3 times by tris-buffered saline buffer with tween 20 (TBST), the protein blots were labeled with the relevant secondary antibodies conjugated with Horseradish Peroxidase (HRP), and signals were visualized using the HyGLO HRP detection kit from Denville (Metuchen, NJ, USA). β-Actin was measured to control for equal loading.

### 3.6. Quantitative Reverse-Transcription-Polymerase Chain Reaction (RT-PCR)

Relative RT-PCR was conducted by TaqMan gene expression master mix from Applied Biosystems (Carlsbad, CA, USA). Briefly, total RNA was isolated from Huh7 cells using the RNeasy kit from Qiagen (Valencia, CA, USA). Reverse transcription was performed to synthesize cDNA by the High Capacity cDNA Reverse Transcription Kit from Applied Biosystems (Carlsbad, CA, USA). The TaqMan assays was performed in an ABI 7300 system using the following profile: 95 °C for 10 min followed by 40 cycles of 15 s at 95 °C and 60 s at 60 °C. MRC2 mRNA levels were normalized to 18 s rRNA mRNA levels in the same samples. The TaqMan probes for MRC2 (Hs00195862_m1) and 18s rRNA (Hs99999901_s1) were both from Applied Biosystems.

### 3.7. Scratch Wound Healing Migration Assay

Huh7 cells were plated into 6-well plates and cultured to more than 90% confluency. Scratch wound were made by a 1000-μL-pipette tip. Images of wounds were taken with a phase-contrast microscope at 0, 24 and 48 h. Cell migration was quantitated by measuring the width of the scratch wounds. Each experiment was repeated three times.

### 3.8. Invasion Assay

Invasion assay was performed using Millicell invasion chambers (8 mm pore size) from Millipore Corp (Billerica, MA, USA). Huh7 cells was grown in serum-free medium overnight and then plated into the upper chamber pre-coated with basement membrane Matrigel. The Huh7 cells were cultured in the upper chamber with serum-free medium and meanwhile the bottom chamber was filled with medium with 20% FBS. These cells were cultured at the normal condition. After 24 h, Huh7 cells remaining in the top surface of Matrigel membrane were swabbed carefully and the membrane was fixed with paraformaldehyde for 10 min. Huh7 cells in the membrane was stained by crystal violet solution and observed under microscope. Five fields were selected randomly to get the mean cell number in each membrane. Each exanimation was repeated six times.

### 3.9. Chromatin Immunoprecipitation (ChIP) Assay

The ChIP assay was performed as described in the previous study [11]. The primers used to detect the MRC2 promoter are designed as following: (forward: 5'-GTCTCAGTCCTGCCCTATGC-3'; reverse: 5'-CGAACTGGGGAGTCAGGATG-3'). The PCR products were separated by electrophoresis, visualized on a 2% agarose gel and compared by the densitometry. Each exanimation was repeated six times.

### 3.10. Statistical Analysis

Differences in MRC2 protein expression in HCC tissues compared to adjacent liver tissues were compared by the Mann–Whitney U test. The relationship between MRC2, TGFβ1 and clinical characteristics of HCCs was assessed using Spearman rank test respectively. Differences between the Kaplan–Meier curves of HCCs with high and low MRC2 expression in HCC tissues in comparison with adjacent liver tissues were evaluated by the log rank test. Statistical significance was accepted at *p* < 0.05. Multivariate analysis was performed using SPSS V17.0 software (SPSS Inc., Chicago, IL, USA) and the PRISM 5 software (GraphPad, La Jolla, CA, USA) was used for other statistical analyses.

## 4. Conclusions

HCC is one of the most frequently occurring tumors worldwide in the last decade. The treatment options with curative intent include radical liver resection, local ablation and liver transplantation at the early stage. Unfortunately, most HCC patients present at the advanced stage, which precludes the application of these curative treatments. As the effect of chemotherapy with conventional cytotoxic agents is unsatisfactory, therapy options for patients with advanced HCC are extremely limited. Tumor metastasis is the key death cause of HCC patients at the advanced stage and thus it is urgent to figure out the molecular mechanism of HCC metastasis and identify the novel therapy targets to prevent HCC metastasis.

MRC2 is a 180-kDa type 1 transmembrane receptor that binds extracellular and intracellular collagens and internalizes them via clathrin-coated pits into early endosomes for lysosomal degradation [23]. There have been accumulating studies reported that MRC2 expression is increased aberrantly in a variety of cancers and associated with poor prognosis. Sulek *et al.* detected MRC2 expression in 112 human squamous cell carcinomas and 19 normal or tumor-adjacent head and neck tissue samples and found that expression of MCR2 was increased in tumor stroma compared with tumor-adjacent connective tissue and related positively with poor differentiation [20]. After analyzing 169 prostate tissue sections including benign prostatic hyperplasia and prostate cancer by immunofluorescence assay, Kogianni *et al.* found that MRC2 expression was clearly increased in both stromal tissues and epithelial tissues of prostate cancer compared to benign prostatic hyperplasia [19]. In glioblastoma multiforme, it was found that MRC2 was over-expressed in tumor tissues and attributed to mediating tumor cells invasion via collagen-containing matrices [24]. In this study, we reported for the first time that MRC2 expression was significantly higher in HCC tissues than that in adjacent liver tissues and associated with intrahepatic metastases and portal vein invasion. After comparing the Kaplan–Meier curves, we found that higher MRC2 expression in HCC tissues predicted worse outcome after liver resection. It was consistent with the results from the Palmieri group [17], reporting that MRC2 was an accuracy prognostic marker for breast cancer metastases. To determine the mechanism of over-expression of MRC2 in HCC, we measured TGFβ1 protein expression in the same HCC samples by IHC and found that there was positive correlation between MRC2 and TGFβ1 expression. TGFβ1 treatment leaded to up-regulation of MRC2 apparently *in vitro*, as well. Moreover, we found 6 potential Smad protein binding sites in the promoter of *MRC2* gene and verified the direct interaction between Smad protein and the MRC2 promoter in HCC cells by ChIP assay. The inhibitor of Smad3 SIS3 inhibited the effect of TGFβ1 treatment on controlling MRC2 transcription in HCC cells dramatically. These indicated eventually that MRC2 over-expression could result from hyperactivation of TGFβ1/Smad3 signaling in HCC. Therefore, it was demonstrated that TGFβ1 regulated MRC2 expression in HCC, which is consistent with the findings in prostate cancer [25] and glioblastoma multiforme [24].

As known to all, TGFβ1 acts as both promoter and repressor via different cell signaling in tumor pathogenesis. To find the role of MRC2 on the TGFβ1 paradox about HCC progression, we treated Huh7 cells with TGFβ1 and found that cell mobility and invasion were enhanced as respected. However, after knockdown of MRC2 by siRNAs, the migration and invasion abilities of Huh7 cells were not affected by TGFβ1 treatment. These data proved strongly that MRC2 took an important part in the oncogenic function of TGFβ1 in HCC.

In summary, this investigation showed that MRC2 expression was aberrantly increased in HCC and associated with intrahepatic metastases and portal vein invasion. Additionally, a high level of MRC2 in HCC tissue predicted a poor outcome after liver resection. It was demonstrated further that MRC2 was regulated directly by TGFβ1/Smad3 and involved in the oncogenic effect of TGFβ1 in HCC. Detection of MRC2 expression in HCC tissues could be very feasible guidance for the diagnosis of HCC invasion and metastasis, also for prediction of outcome after liver resection.
